# Microstructure and shear properties of ultrasonic-assisted Sn2.5Ag0.7Cu0.1RExNi/Cu solder joints under thermal cycling

**DOI:** 10.1038/s41598-021-85685-6

**Published:** 2021-03-18

**Authors:** Jianguo Cui, Keke Zhang, Di Zhao, Yibo Pan

**Affiliations:** 1 School of Materials Science and Engineering, Henan University of Science and Technology, Luoyang, 471023 China; 2 Provincial Ministerial Co-construction of Collaborative Innovation Center for Non-ferrous Metal New Materials and Advanced Processing Technology, Luoyang, 471023 China

**Keywords:** Engineering, Materials science

## Abstract

Through ultrasonic wave assisted Sn2.5Ag0.7Cu0.1RExNi/Cu (x = 0, 0.05, 0.1) soldering test and − 40 to 125 °C thermal shock test, the microstructure and shear properties of Sn2.5Ag0.7Cu0.1RExNi/Cu solder joints under thermal cycling were studied by the SEM, EDS and XRD. The results show that the Sn2.5Ag0.7Cu0.1RExNi/Cu solder joints with high quality and high reliability can be obtained by ultrasonic assistance. When the ultrasonic vibration power is 88 W, the ultrasonic-assisted Sn2.5Ag0.7Cu0.1RE0.05Ni/Cu solder joints exhibits the optimized performance. During the thermal cycling process, the shear strength of ultrasonic-assisted Sn2.5Ag0.7Cu0.1RExNi/Cu solder joints had a linear relationship with the thickness of interfacial intermetallic compound (IMC). Under the thermal cycling, the interfacial IMC layer of ultrasonic-assisted Sn2.5Ag0.7Cu0.1RExNi/Cu solder joints consisted of (Cu,Ni)_6_Sn_5_ and Cu_3_Sn. The thickness of interfacial IMC of ultrasonic-assisted Sn2.5Ag0.7Cu0.1RExNi/Cu solder joints was linearly related to the square root of equivalent time. The growth of interfacial IMC of ultrasonic-assisted Sn2.5Ag0.7Cu0.1RExNi/Cu solder joints had an incubation period, and the growth of IMC was slow within 300 cycles. And after 300 cycles, the IMC grew rapidly, the granular IMC began to merge, and the thickness and roughness of IMC increased obviously, which led to a sharp decrease in the shear strength of the solder joints. The 0.05 wt% Ni could inhibit the excessive growth of IMC, improve the shear strength of solder joints and improve the reliability of solder joints. The fracture mechanism of ultrasonic-assisted Sn2.5Ag0.7Cu0.1RExNi/Cu solder joints changed from the ductile–brittle mixed fracture in the solder/IMC transition zone to the brittle fracture in the interfacial IMC.

## Introduction

Today, with the rapid development of electronic information technology and the enhancement of people’s sense of environmental protection, various electronic devices are developing to integrated modules, and the internal solder joints are becoming denser, which raises higher requirements for the quality and reliability of solder joints. Therefore, the development of environmentally friendly lead-free solder joints and high-reliability halogen-free solder joints has become a research hotspot in this field^1–3^.

Among many lead-free solders, SnAgCu-based solders, especially SnAgCuRE-based solders are considered to be one of the best substitutes for Sn–Pb solder^4–6^. However, the wettability of SnAgCu-based solder alloys still requires improvement for the the higher requirements of electronic packaging. In addition, the solder joints will deteriorate the reliability under harsh conditions of service, such as mechanical shocks, thermal cycling and electromigration^7–9^. Thus, it is necessary to develop a new method to improve the solder quality and the solder joints reliability under halogen-free conditions. There are two approaches to improve the solder performance and solder joints reliability. One is to improve the soldering process, such as applying ultrasonic vibration; the other is to improve the lead-free solder wettability by adding trace elements (such as Ni, RE)^8, 10–16^. Ultrasonic is considered as an effective method to remove the surface oxide film and improve the solder wettability to obtain high quality solder joints, which has become a research hotspot^2,6,14^. Ji et al.^17^ investigated the effect of the ultrasonic vibration on the microstructure and properties of lead-free solder joints, which showed that the solder joints possessed a refined microstructure. Terada et al.^15^ studied the effect of Ni on Sn-0.7Cu solder and solder joint. The results showed that adding Ni can refine the microstructure and improve the stabilities of interfacial structures in Sn-0.7Cu solder alloys and joints. Zhao et al.^10^ reported that Ce decreased the interfacial IMC grain size and the interfacial IMC thickness. Pr was reported to refine the Sn-9Zn-0.5 Ga solder microstructures^12^. La and Ce can improve the creep-rupture life of solder joints remarkably^13^.

As we all know, the solder joints reliability has an important relationship with the morphology and size of the interfacial intermetallic compound (IMC)^18,19^. So it is extremely necessary to understand the growth behavior of interfacial IMC. The results show that the shear strength of prismatic IMC is higher than that of f scalloped grains^18^. Zhang et al.^7^ studied interface reaction mechanism during thermal cycling and isothermal aging. The results show that the morphology of IMCs was gradually changed from scallop-like to planar-like, and the thickness of different IMCs evolved with the increasing of aging time. Teo et al.^20^ numerically and experimentally studied morphological evolution of IMC subjected to thermal cycling loading. In this study, the IMC grains ripened into large hemispherical-type and spheroidal-type morphologies and spalled into the solder joint during temperature cycling. For needle-type grains, the stress was concentrated at the tip of the IMCs, while the maximum shear stress was redistributed to the roots of the spheroidal-type grains. Cao et al.^21^ further studied the IMC microstructure and shear strength of Sn2.5Ag0.7Cu0.1RExNi/Cu solder joints under thermal cycling and found the fracture pathway changed from solder seam with ductile fracture to seam/IMC with ductile–brittle mixed fracture from 100 to 500 cycles.

From the above literatures, we can reasonably believe that the ultrasonic-assisted soldering process can refine the microstructure of solder. However, up to now, there are few reports on the reliability of ultrasonic-assisted solder joints in harsh environments^6^. Therefore, in this study, we investigated the characteristics of ultrasonic-assisted Sn2.5Ag0.7Cu0.1RExNi/Cu lead-free solder joints under thermal cycling. And it reveals the effect of thermal cycling on the morphology of the interfacial IMC and shear properties of ultrasonic-assisted Sn2.5Ag0.7Cu0.1RExNi/Cu (x = 0, 0.05, 0.1) solder joints in terms of the IMC sizes, shear strength and fracture morphology, and provides a new way for the development of high reliability soldering under harsh conditions of service.

## Experimental procedure

Sn2.5Ag0.7Cu0.1RExNi (x = 0, 0.05, 0.1) solder alloys were prepared by induction melting of high-purity raw materials (99.9 wt% Sn, 99.9 wt% Ag, 99.9 wt% Cu, 99.9 wt% Ni and RE including La and Ce) in a vacuum furnace^6^. First, intermediate alloys of Cu and RE were obtained in a melting oven at a vacuum degree of 5 × 10^−3^ Pa. Second, Sn2.5Ag0.7Cu0.1RExNi (x = 0, 0.05, 0.1) solder alloys were prepared at the same way.

The solder alloys were rolled to sheets with 0.1 mm thickness for further use after melting. Finally, the solder alloys were prepared into sheets with 10 mm in width, 20 mm in length and 0.1 mm in thickness. In this study, the dimensions of the Cu substrate (99.99%) were 75 mm × 20 mm × 2 mm. Before reflowing, the copper samples were carefully ground by using wet emery papers and then cleaned in methanol alcohol and water.

**Figure 1:**
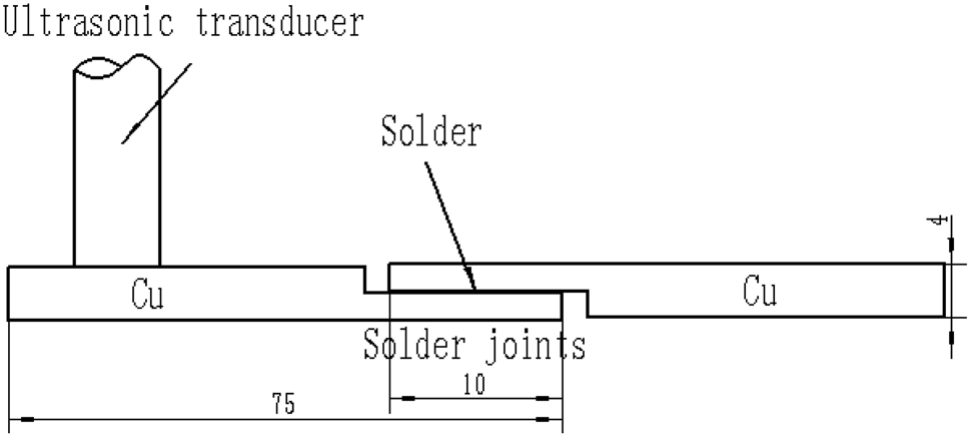
Geometry of the dimension of solder joints samples and application USW to samples (Unit: mm).

A piece of solder sheet with dimensions of 10 × 20 × 0.1  mm^3^ was placed between the two copper substrates before soldering (see Fig. 1). The commercial CX600 was adopted as flux in the soldering process, and the corrosivity of the flux was low. We obtained the solder joints by using the special soldering furnace. It can provide ultrasonic vibration in the soldering process.

**Figure 2:**
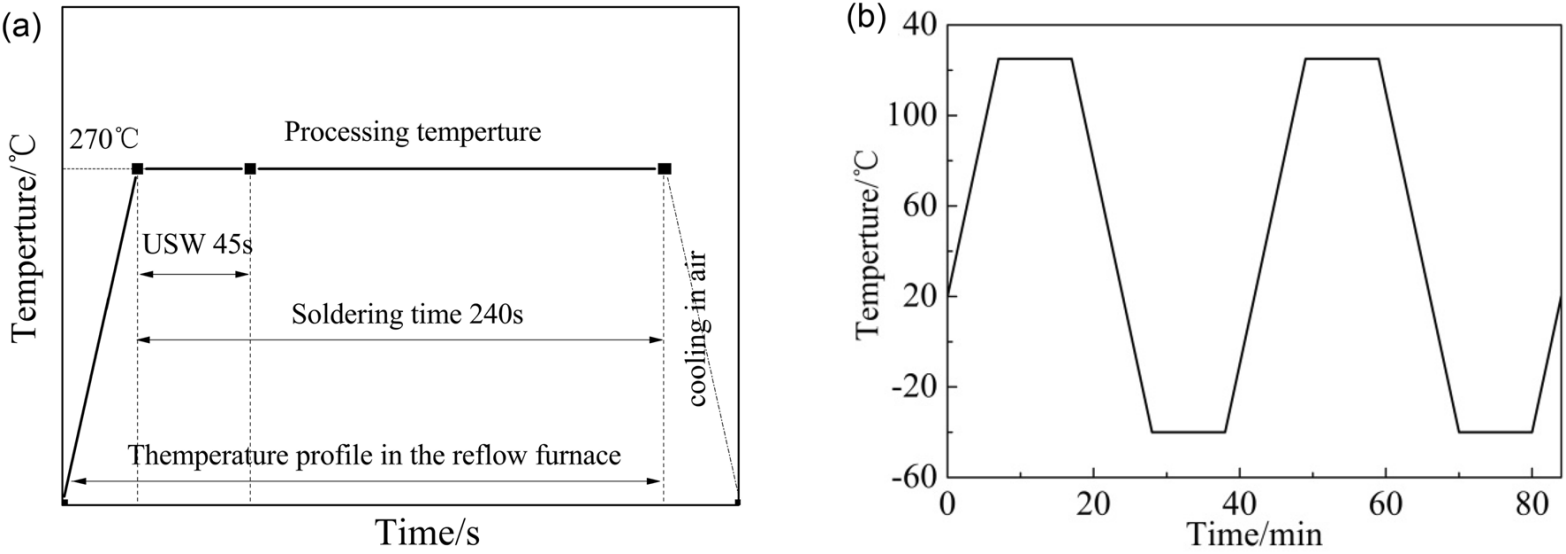
Parameters of the soldering process and thermal cycling: (**a**) processing parameters for the solder joints, (**b**) thermal cycling curve.

The soldering temperature and the soldering time were 270 ℃ and 240 s, respectively (see Fig. 2a)^12^. Once the solder joints samples had been maintained at 270 ℃, a 20-kHz ultrasonic transducer was laid up directly on the top of the solder joints samples (see Fig. 1). The solder joints samples were exposed using the ultrasonic power 0 W, 22 W, 44 W, 66 W, 88 W and 110 W, respectively. The solder joints samples were exposed to ultrasonic vibration (USV) for 45 s. When soldering time met the requirements, the solder joints samples were removed from the oven and cooled in air. Then the best ultrasonic vibration power were selected for soldering experiment.

Additionally, the solder joints samples with optimized performance were tested in the furnace chamber for cycling temperature reliability. The temperature range was – 40 ℃ to + 125 ℃, which conformed to IPC-9701A standard^21,22^ (dwell time at each temperature extreme: 10 min; ramp rate: ~ 15 ℃/min) (see Fig. 2b) for 100 cycles, 300 cycles, 500 cycles, 700 cycles and 1000 cycles. When thermal cycles met the requirements, the solder joints samples were taken out of the furnace chamber and cooled in air. The exposure time at 125 ℃ is equal to the total dwell time spent at the high-temperature soak period for the thermal cycling profiles^13^.

For microstructural observation, the reflowed and thermally cycled samples were first cut in the center, and then ground and polished. The etching with 4% HNO_3_ in alcohol was applied for further observation. The cross-sectional structure of samples was observed by the JSM-5610LV Scanning Electron Microscope (SEM). Meanwhile, the composition analysis was evaluated using an Energy Dispersive Spectroscopy (EDS). For the top-view observation of the interfacial IMC, the deep etching was performed using 20% HCL in alcohol to remove the Sn of the solder joints sample and expose the interfacial IMC. The shear strength of the samples was carried out by a tensile testing machine at room temperature (rate: 1 mm/min). The studies of fracture morphology characterization were also observed by using the SEM on the fracture surface of Sn2.5Ag0.7Cu0.1RExNi (x = 0, 0.05, 0.1) solder joints samples, to investigate the failure mechanisms. The phase composition of the shear fracture morphology was then analyzed by D8-ADVANCE X-ray Diffractometer (XRD) fitted with Cu Kα radiation with the range of 20–90°.

**Figure 3:**
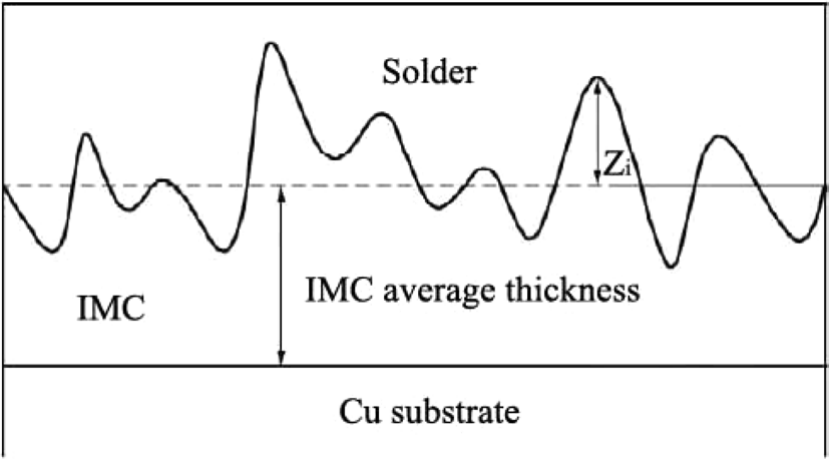
Schematic of Rrms at the solder joint interface.

Figure 3 displays a schematic diagram of roughness (*R*_*rms*_) of the interfacial IMC. The area of IMC layers was measured by using AutoCAD software, aimed at measuring average thickness (d) of the interfacial IMC layers, which was expressed by Eq. (1). The roughness (*R*_*rms*_) of the interfacial IMC could be calculated by Eq. (2)^23^

1 $$d = {\text{A}}/{\text{L}}$$

where the *d* is the average thickness of the interfacial IMC, the A is the area of the interfacial IMC, the L is the length of the coverage.

2 $$R_{rms} = \sqrt {\frac{{\sum\nolimits_{i = 1}^{N} {Z_{i}^{2} } }}{N}}$$

where the *N* is the data number of *Z*_*i*_ (N ≥ 15).

## Results and discussion

### Interfacial microstructure of solder joints

**Figure 4:**
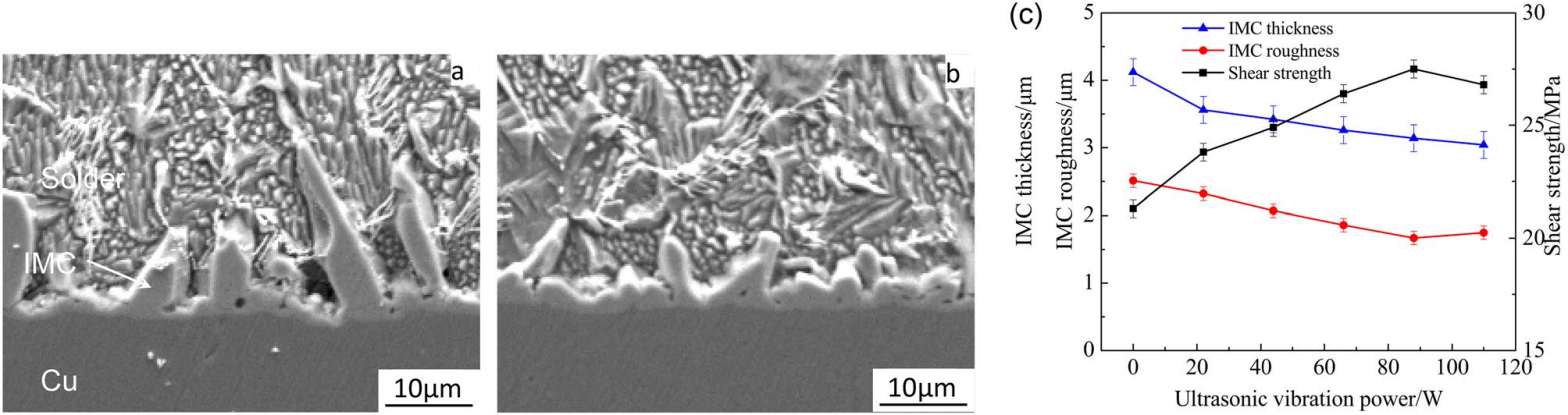
Effects of ultrasonic vibration power on ultrasonic-assisted Sn2.5Ag0.7Cu0.1RE0.05Ni/Cu solder joints: (**a**) 0 W, (**b**) 88 W, (**c**) IMC thickness, IMC roughness and shear strength of the solder joints.

The SEM images of interfacial microstructure of ultrasonic-assisted Sn2.5Ag0.7Cu0.1RExNi/Cu solder joints are shown in Fig. 4a,b, respectively. It can be seen that solder joints consist of three colonies: solder seam, Cu substrate and IMC layer (see Fig. 4a,b). The base metal area is copper matrix, and the interfacial area is “scallop like” IMC layer. The solder seam is composed of primary β-Sn, reticular eutectic structure. And combined with the Sn–Ag–Cu ternary phase diagram, the eutectic structure consists of binary eutectic structure (β-Sn + Cu_6_Sn_5_, and β-Sn + Ag_3_Sn) and ternary eutectic structure (β-Sn + Cu_6_Sn_5_ + Ag_3_Sn)^6^. The IMC morphology of ultrasonic-assisted Sn2.5Ag0.7Cu0.1RExNi/Cu solder joints with ultrasonic vibration power 88 W is shown in Fig. 4b. The morphology of IMC layer is more flat and smooth. Figure 4c shows the effects of ultrasonic vibration power on ultrasonic-assisted Sn2.5Ag0.7Cu0.1RE0.05Ni/Cu solder joints. With the increase of ultrasonic vibration power, the interfacial IMC thickness and roughness decreased gradually, which increased the shear strength of the solder joints. When the ultrasonic vibration power is 88 W, the ultrasonic-assisted Sn2.5Ag0.7Cu0.1RE0.05Ni/Cu solder joints exhibits the optimized performance (increases 29.1% comparing to the solder joints without ultrasonic), which was consistent with the previous studies^6,14^.

**Figure 5:**
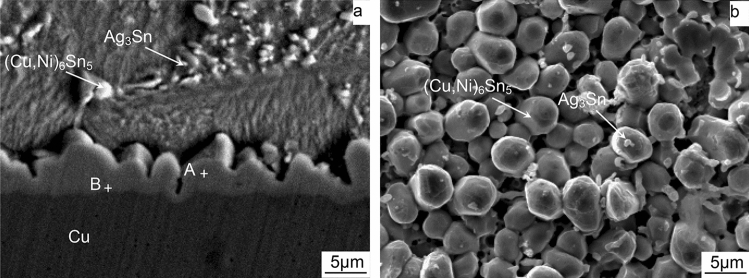
The interfacial microstructure of Sn2.5Ag0.7Cu0.1RE0.05Ni/Cu solder joints and EDS analysis (100cycles) (**a**) cross-sectional, (**b**) top view.

The cross-sectional and top-view SEM images of interfacial microstructure of ultrasonic-assisted Sn2.5Ag0.7Cu0.1RExNi/Cu solder joints under thermal cycling are shown in Fig. 5a,b, respectively. The energy spectrum analysis results of points A and B in Fig. 5a are shown in Table 1. As can be seen from Fig. 5a, there are two continuous IMC layers, one is near the side of the solder seam, and the other is near the Cu matrix side. According to the analysis results, the IMC layer on the top is (Cu,Ni)_6_Sn_5_, and the IMC layer at the bottom is Cu_3_Sn, which is consistent with previous studies^24^. This result occurred because the Cu and Ni atoms possessed the same crystal structure. Thus some Ni atoms substituted Cu sites in the IMC lattice in the solidification process. It can be seen that the thickness of (Cu,Ni)_6_Sn_5_ IMC layer is thicker than that of Cu_3_Sn IMC layer. The IMC overlooking morphology of ultrasonic-assisted Sn2.5Ag0.7Cu0.1RE/Cu solder interface is shown in Fig. 5b. The crystal structure of the (Cu,Ni)_6_Sn_5_ IMC layer is “ellipsoid”, and the white micro-particles on the (Cu,Ni)_6_Sn_5_ grain surface are Ag_3_Sn particles formed in the soldering seam (see Fig. 5b). It showed that these nanoscale Ag_3_Sn could inhibit the growth of interfacial IMC^25^.

**Table 1:** EDS analysis results of solder joint interface shown in Fig. 5a.

Point	Mole fraction/%			
	Sn	Cu	Ni	Ag
A	43.58	54.21	2.21	–
B	24.63	75.37	–	–

### IMC growth behavior

**Figure 6:**
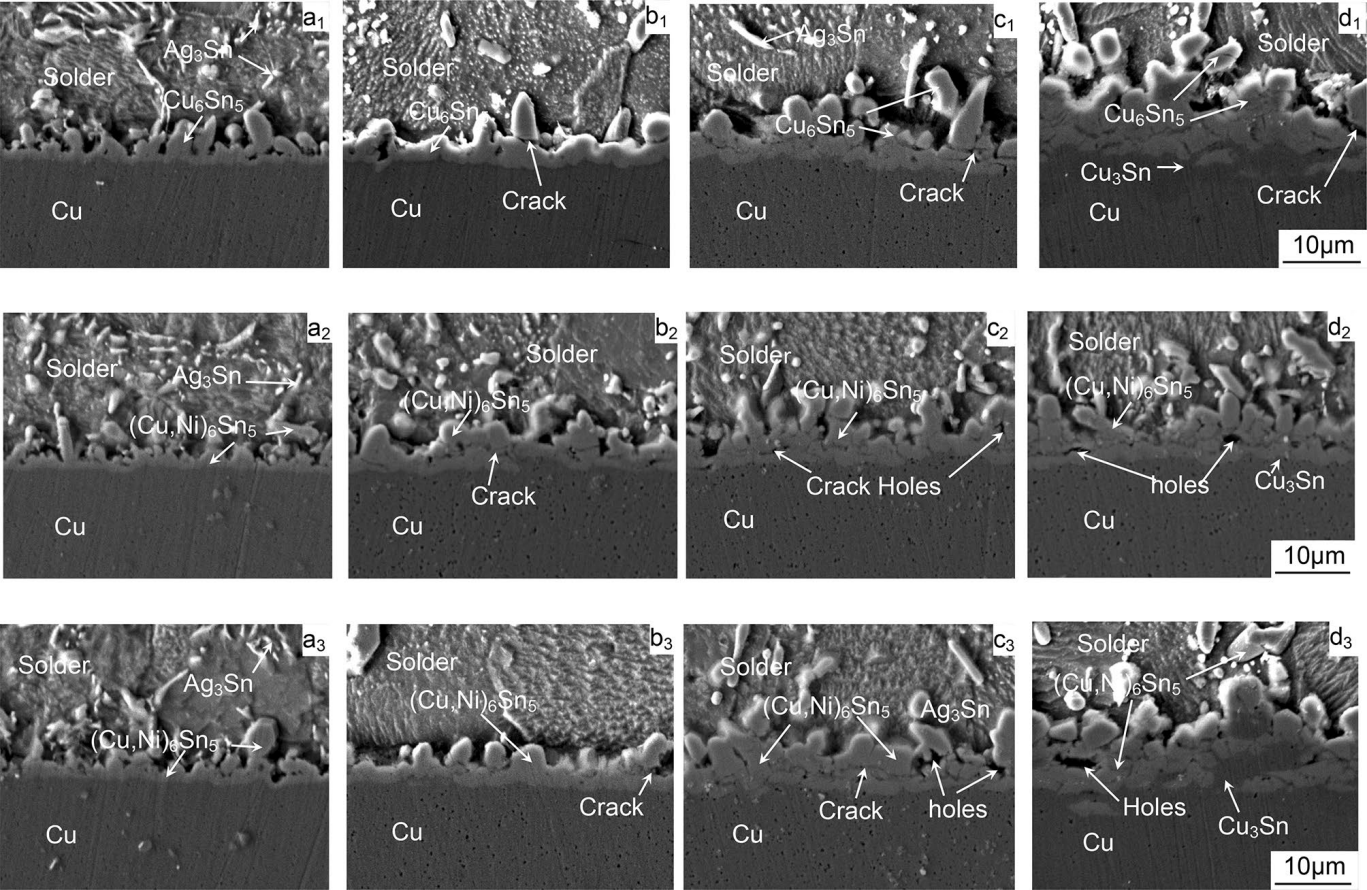
The SEM images of IMC of Sn2.5Ag0.7Cu0.1RExNi/Cu solder joints (**a**_**1**_–**d**_**1**_) 0Ni, (**a**_**2**_–**d**_**2**_) 0.05Ni, (**a**_**3**_–**d**_**3**_) 0.1Ni, (**a**) cycle, (**b**) 300 cycles, (**c**) 700 cycles, (**d**) 1000 cycles.

**Figure 7:**
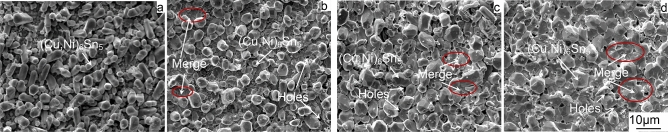
Top-view of IMC of Sn2.5Ag0.7Cu0.1RExNi/Cu solder joints after 0, 300, 700, 1000 thermal cycles. (**a**) 0 cycle, (**b**) 300 cycles, (**c**) 700 cycles, (**d**) 1000cycles.

**Figure 8:**
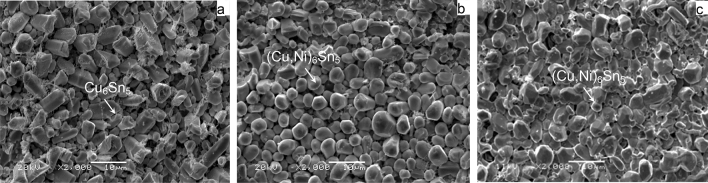
Top-view of IMC at the interface of Sn2.5Ag0.7Cu0.1RExNi/Cu after 500 thermal cycles: (**a**) 0 Ni, (**b**) 0.05 Ni, (**c**) 0.1 Ni.

During soldering reflow, the molten solder reacts with the Cu substrate forming a layer of intermetallic compound (IMC). The presence of interfacial IMC indicates that a good metallurgical bond has been formed, but their inherent brittle nature and tendency to concentrate stress and create defects often undermine reliability under stress conditions, such as thermal cycling. The excessive growth of intermetallic compounds may be detrimental to the reliability of the solder joints. Generally speaking, the layer of interfacial IMC has a remarkable effect on the reliability of solder joints^20,26^. Therefore, it is necessary to study the morphology and size of the interfacial IMC under thermal cycling. The cross-sectional and top-view SEM images of the interfacial IMC of ultrasonic-assisted Sn2.5Ag0.7Cu0.1RExNi/Cu (x = 0, 0.05, 0.1) solder joints after thermal cycles are shown in Figs. 6,7 and 8.

The cross-sectional images of the interfacial IMC of ultrasonic-assisted Sn2.5Ag0.7Cu0.1RExNi/Cu (x = 0, 0.05, 0.1) solder joints under various thermal cycles are shown in Fig. 6. The interfacial IMC of as-soldered joints as the control sample (Fig. 6a) was only (Cu,Ni)_6_Sn_5_ IMC layer, and the interfacial IMC was “scallop like”. However, the interfacial IMC of solder joints under various thermal cycles (Fig. 6b–d) was composed of (Cu,Ni)_6_Sn_5_ IMC layer and Cu_3_Sn IMC layer compared to Fig. 6a. In addition, the interfacial IMC of solder joints under various thermal cycles (Fig. 6b–d) was “lamellar”. From the top-view images Fig. 7, it can be found that the IMC grains were a short rod-like which was in accordance with the corresponding images of Fig. 6. With the increasing the thermal cycles, the interfacial IMC layer of solder joints gradually grew and thickened, and the morphology of the interfacial IMC gradually changed from “scallop like” to “lamellar”^7,15^. And the size of IMC grains increased with the increase of the thermal cycles (Fig. 7). Meanwhile the morphology of the Ag_3_Sn changed from needle-like (Fig. 6a) to short rod-like (Fig. 6b–d). After 300 cycles, the interfacial IMC of solder joints (Fig. 6b–d) began to exist microcracks and microvoids, and the interfacial IMC thickness increased. As shown the top-view images (Fig. 7b–d), it could be found that the IMC grains began to merge together^15^. After 700 cycles, the thickness of the interfacial IMC was obviously increased (see Fig. 6c,d). And the spalling behavior of the interfacial IMC in the process of thermal cycling was also been seen, which might be caused by the cyclic shear stresses and strains induced during temperature cycling^7^. After 1000 cycles, there were many microcracks and microvoids in IMC layer (Fig. 6d).

**Figure 9:**
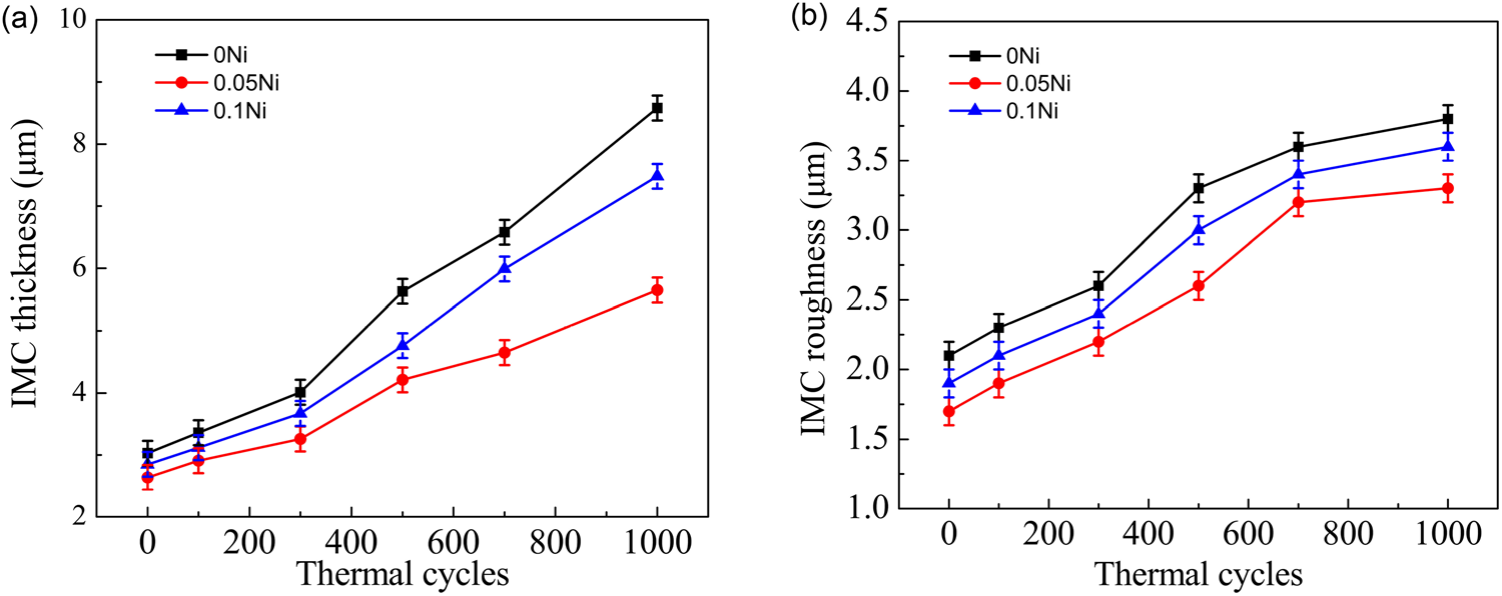
The interfacial IMC thickness and roughness of Sn2.5Ag0.7Cu0.1RExNi/Cu solder joints. (**a**) The interfacial IMC thickness, (**b**) the interfacial IMC roughness.

Figure 9 shows the average thickness (d) and roughness (R_rms_) of the interfacial IMC of ultrasonic-assisted Sn2.5Ag0.7Cu0.1RExNi/Cu solder joints under various thermal cycles. With increasing thermal cycles, the d and R_rms_ of the interfacial IMC increased. After 1000 cycles, the d of the interfacial IMC of Sn2.5Ag0.7Cu0.1RExNi/Cu (x = 0, 0.05, 0.1) solder joints increased by 8.6 μm, 5.7 μm and 7.5 μm, respectively (increased 186%, 114% and 162%, respectively). Correspondingly, the R_rms_ of the interfacial IMC of Sn2.5Ag0.7Cu0.1RExNi/Cu (x = 0, 0.05, 0.1) solder joints increased by 3.8 μm, 3.3 μm and 3.6 μm, respectively (increased 80%, 94% and 89%, respectively). Under the same thermal cycling condition, when the addition of Ni element was 0.05 wt%, the d and R_rms_ of interfacial IMC were the smallest, which was consistent with the rule of Fig. 6. And, the d of the interfacial IMC was approximately linear with the thermal cycles. Moreover, the slope of the solder joints with 0.05 wt% Ni was obviously lower than that of the others, which indicated that Ni element has a significant effect on the growth of the interfacial IMC^27–29^. During the soldering and thermal cycling process, the Sn from the filler metal and the Cu mainly from the Cu matrix reacted to form the Cu-Sn IMC layer. Because Ni can react with Cu and Sn to form Cu–Ni–Sn IMC. It might be due to the Ni as a pinning to retard the diffusion of Sn atoms to the interface^8^. Therefore, the addition of Ni can effectively inhibit the growth of the interfacial IMC.

The kinetics of the interfacial IMC growth can be diffusion controlled to interfacial-reaction controlled^7^. The thickness the IMC layer is a function of the time can be calculated using the following Eq. (3)^7^:

3 $$X_{t} = {\text{ X}}_{0} + \sqrt {{\text{Kt}}}$$

where *X*_*t*_ is the thickness of the interfacial IMC layer at equivalent aging time *t*, X_0_ is the initial thickness of the interfacial IMC layer after soldering, and K is the growth rate of the interfacial IMC. The K for the interfacial IMC is also the diffusion coefficient, which is a function of temperature.

**Figure 10:**
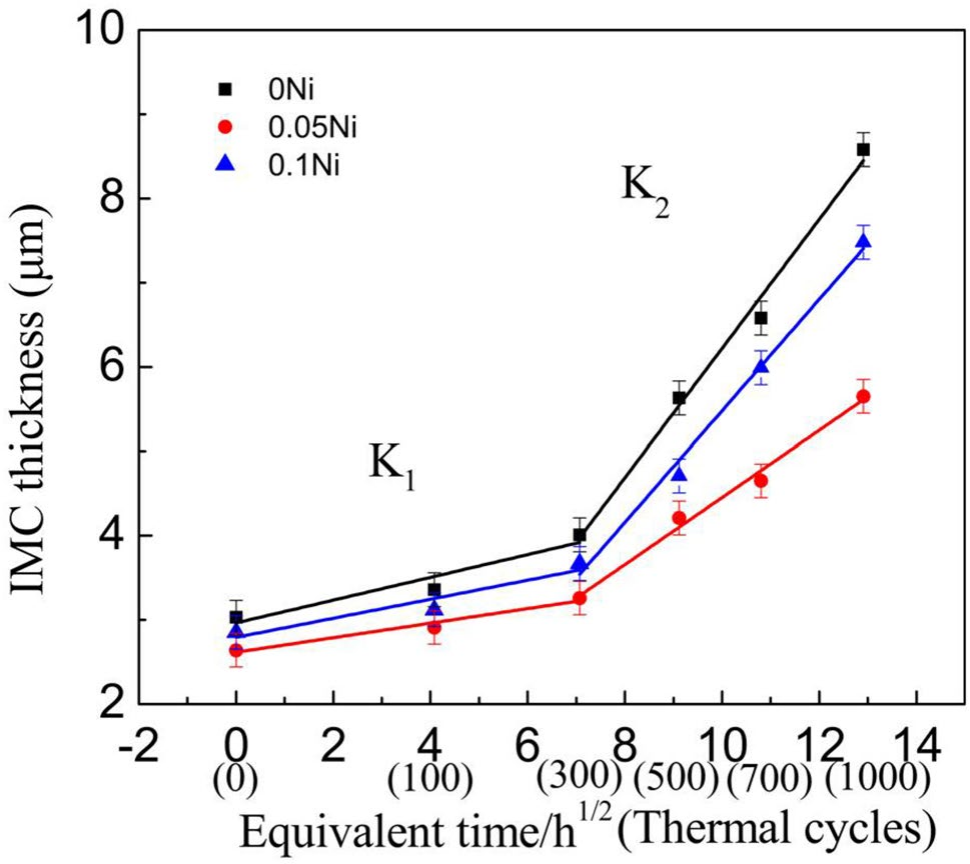
Relationship between IMC thickness and the square root of equivalent time.

Figure 10 shows the relationship between of the average thickness (d) of the interfacial IMC layer and the equivalent aging time (t) for thermal cycling. For Cu_3_Sn IMC, the thickness of interfacial Cu_3_Sn IMC layer was close to 1 μm under 1000 thermal cycles. It indicated that the growth interfacial IMC of the solder joints was mainly (Cu,Ni)_6_Sn_5_ IMC under all conditions (as-soldered and thermal cycling). The average thickness of the interfacial IMC layer had a linear relationship with the square root of the equivalent aging time and the K_2_ of IMC was faster than the K_1_ of IMC. And it indicated the IMC grew faster in 300–1000 cycles, which was consistent with the changes in interfacial IMC morphology of solder joints shown in Fig. 6. For IMC, with Ni additions, the K of interfacial IMC layer decreased. It indicated that the growth rate of interfacial IMC of the solder joints with Ni grew slower than that of the monolithic Sn2.5Ag0.7Cu0.1RE/Cu solder joints under all conditions (as-soldered and thermal cycling).The results showed that the addition of an appropriate amount of Ni could inhibit the excessive growth of IMC, this result was consistent with the above analysis.

### Shear strength of solder joints

Additionally, solder joints not only were an electrical integration, but also were subjected to mechanical loading in the service conditions. Therefore, it is very important to test the mechanical properties of the solder joints. In order to better simulated actual service conditions, the shear tests of solder joints samples were carried out under thermal cycling. And, we investigated the influence of thermal cycling and Ni on the shear strength of solder joints.

**Figure 11:**
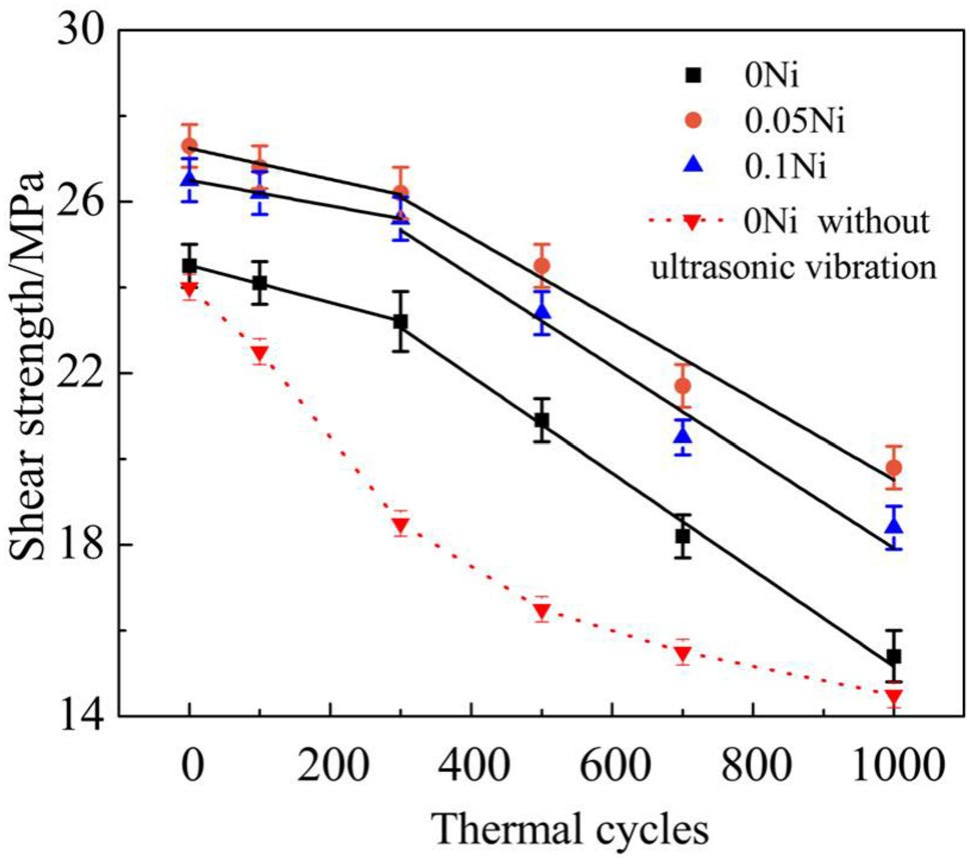
Shear strength of solder joints.

**Figure 12:**
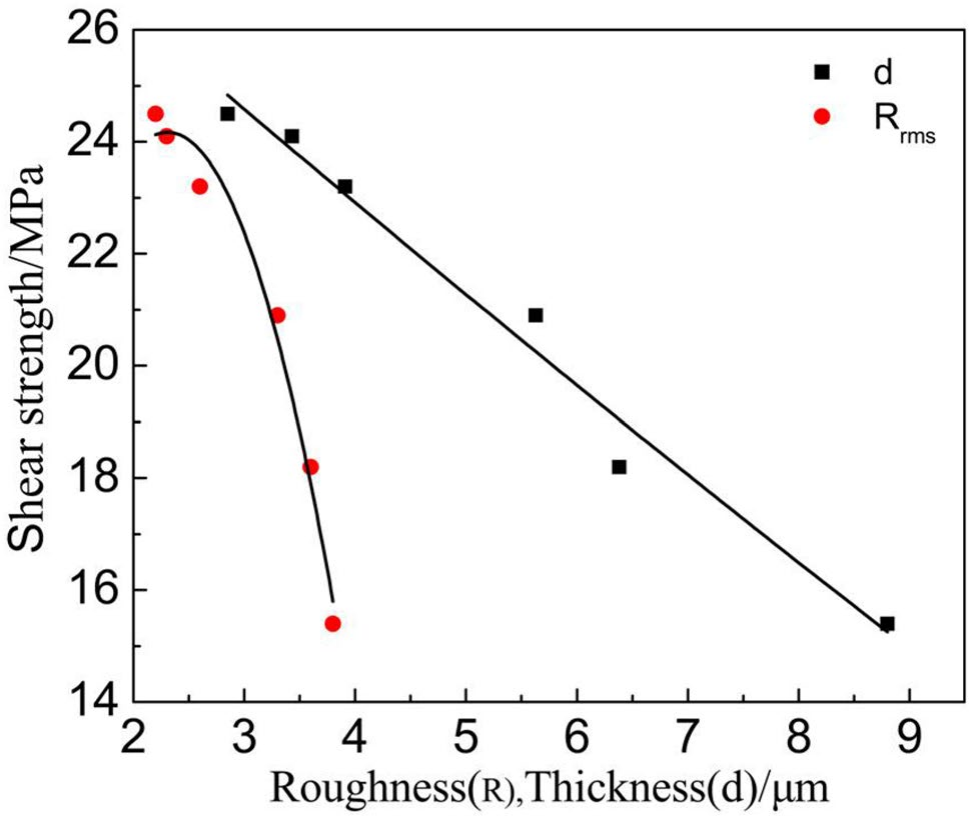
Relationship between shear strength and *d, R*_*rms*_.

Figure 11 shows the shear strength of ultrasonic-assisted Sn2.5Ag0.7Cu0.1RExNi/Cu solder joints under various thermal cycles. The shear strength of Sn2.5Ag0.7Cu0.1RE/Cu solder joints without ultrasonic as the control sample (Fig. 11) was always smallest under different thermal cycles, which indicated that ultrasonic improved the thermal cycling reliability of solder joints. For ultrasonic-assisted solder joints, when the thermal cycling was within 300 cycles, the growth of IMC was slow, the thickness (d) of IMC was close to 4 μm and the shear strength of solder joints decreased slowly. After 300 cycles, the interfacial IMC layer grew rapidly, and the thickness (d) and roughness (R_rms_) of IMC increased rapidly, which led to the rapid decrease of the shear strength of the solder joints (see Figs. 10 and 11). This was consistent with the linear relationship IMC thickness between and the shear strength in Fig. 12. With increasing thermal cycles, the shear strength decreased. When the thermal cycles increased to 300 cycles, the shear strength of the Sn2.5Ag0.7Cu0.1RExNi/Cu (x = 0, 0.05, 0.1) solder joints decreased by 23.2 MPa, 26.8 MPa and 25.6 MPa, respectively (decreases of 5.6%, 1.8% and 3.4%, respectively). When the thermal cycles increased to 1000 cycles, the shear strength of the Sn2.5Ag0.7Cu0.1RExNi/Cu (x = 0, 0.05, 0.1) solder joints decreased by 15.4 MPa, 19.8 MPa and 18.6 MPa, respectively (decreases of 33.6%, 27.5% and 30.6%). And, the shear strength of solder joints was linearly correlated with the thermal cycles. In additon, the slope of the fitting line of the former was significantly lower than that of the latter, which showed that 300–1000 cycles had a significant effect on the shear strength of the solder joints. It could also be seen from Fig. 11 that when the content of Ni was 0.05 wt%, the shear strength of ultrasonic-assisted Sn2.5Ag0.7Cu0.1RExNi/Cu solder joints was the highest under the same thermal cycles. And the slope of solder joints with Ni was slower than that of solder joints without Ni. The results showed that the addition of an appropriate amount of Ni could inhibit the excessive growth of IMC, improve the shear strength of solder joints and delay the decrease of shear strength of solder joints under thermal cycling, thus improving the thermal cycling reliability of solder joints.

Figure 12 shows the relationships among the d, Rrms of the interfacial IMC ((Cu,Ni)_6_Sn_5_ IMC and Cu_3_Sn IMC) and shear strength of ultrasonic-assisted Sn2.5Ag0.7Cu0.1RExNi/Cu solder joints. The average thickness (d) of the interfacial IMC had a linear relationship with the shear strength of the solder joints. It indicated that the shear strength of the solder joints was mainly affected by the thickness (d) of the interfacial IMC, which was in accordance with the results of the literature^30,31^. The results also showed that the growth of IMC was related to the accumulation of high temperature stage in the thermal cycling process.

**Figure 13:**
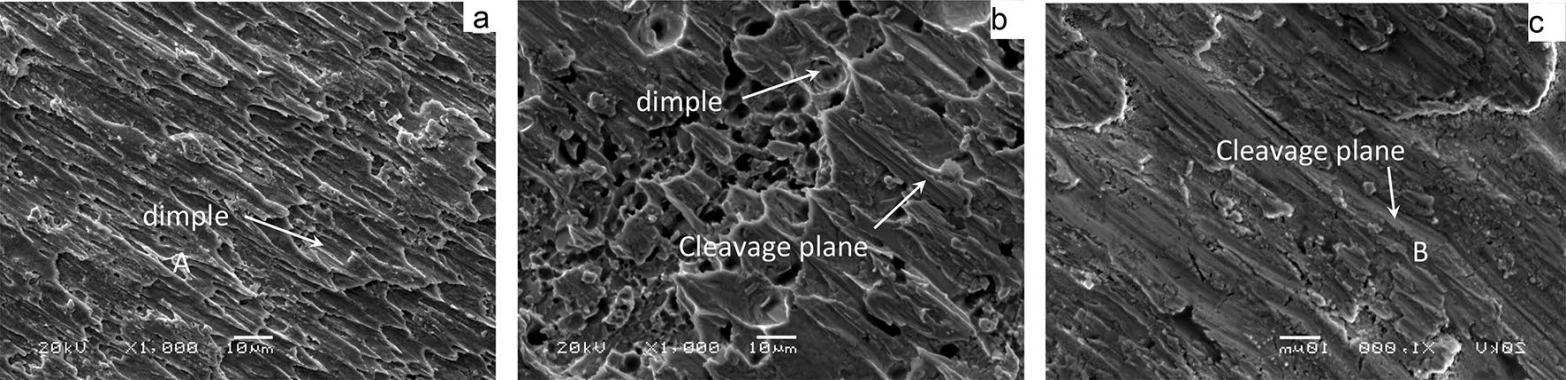
Shear-fractured morphology of Sn2.5Ag0.7Cu0.1RE0.1Ni/Cu solder joints after 0, 300, 1000 thermal cycles (**a**) 0cycles, (**b**) 300cycles, (**c**) 1000 cycles.

**Figure 14:**
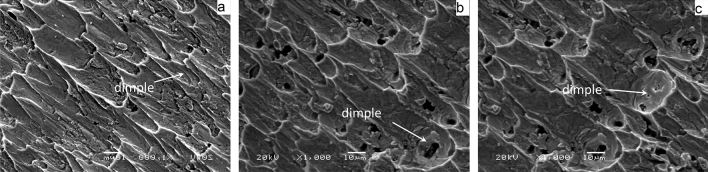
Shear-fractured morphology of solder joints after 500 thermal cycles (**a**) 0Ni, (**b**) 0.05Ni, (**c**) 0.1Ni.

**Figure 15:**
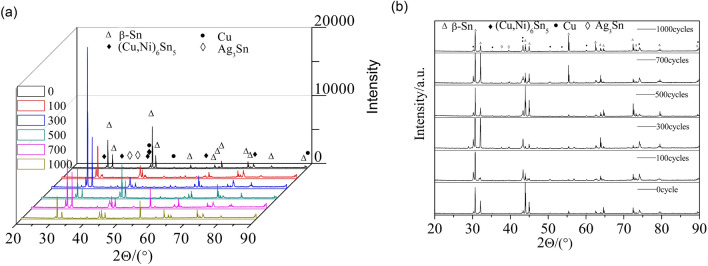
XRD analysis of shear-fractured morphology of Sn2.5Ag0.7Cu0.1RExNi/Cu solder joints based on (**a**) actual intensity, (**b**) intensity in arbitrary.

**Figure 16:**
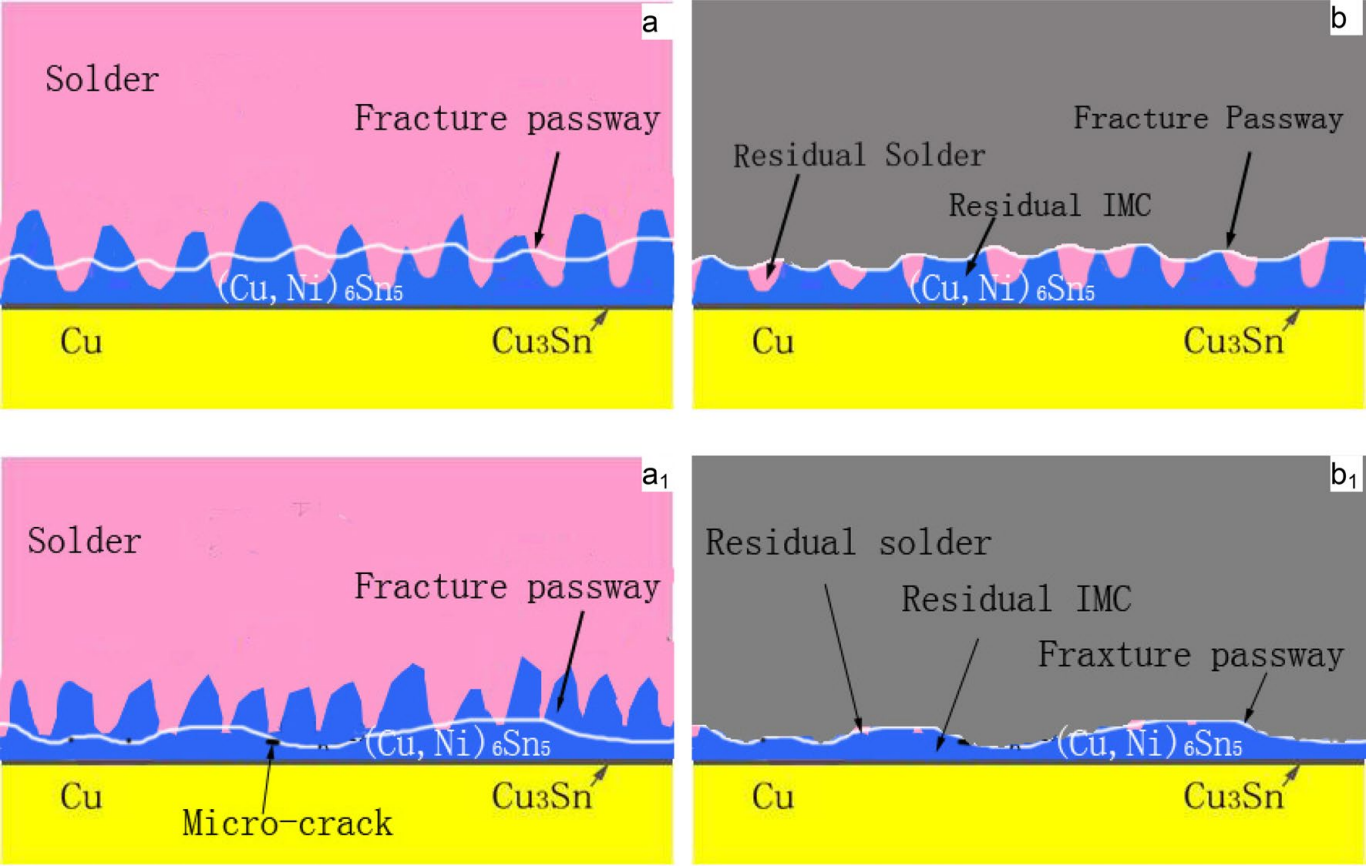
Failure modes of solder joints: (**a**,**b**) solder/IMC mode, (**a**_**1**_,**b**_**1**_) IMC mode. (**a**,**a**_**1**_) Fracture pathway, (**b**,**b**_**1**_) Side view of shear fracture.

Figure 13 shows the shear fracture morphology of ultrasonic-assisted Sn2.5Ag0.7Cu0.1RExNi/Cu solder joints fabricated under various thermal cycles. The shear fracture morphology of the control sample had parabolic dimples and a small amount of cleavage planes (Fig. 13a), which was the ductile–brittle mixed fracture mode. With increasing the thermal cycles, the number of cleavage planes increased. Meanwhile the number of parabolic dimples decreased, which indicated that the proportion of the ductility region of the shear fracture morphology decreased with increasing thermal cycles (Figs. 13b,c, 14c). From the shear fracture morphology in Fig. 13b, we could see that the solder joints under thermal cycling was brittle–ductile fracture mode. The shear fracture morphology of solder joints was mainly cleavage planes (Fig. 13c), which was the brittle fracture mode. The composition of the fracture morphology was measured by EDS analysis. Table 2 were the EDS of the “A” and “B” areas in Fig. 13. And the results showed that the parabolic dimples consisted of mainly Sn and small amount of Ag and Cu and the cleavage plane contained mainly the Sn and Cu. The atomic ratio of Cu to Sn was close to 6:5, and it might be (Cu,Ni)_6_Sn_5_. The results showed that with increasing thermal cycles, the fracture mechanism of the solder joints changed from the ductile–brittle mixed fracture mode with cleavage of the interfacial IMC and dimpling of the solder seam to brittle fracture with cleavage of interfacial IMC and cleavage of the solder seam. In addition, the crack pathway changed from the interfacial transition zone which was consisted of the interfacial IMC layer and the solder seam to the interfacial IMC layer zone (see Fig. 16, the transition from fracture model of solder/IMC zone to IMC fracture model^32,33^). Figure 14 shows the fracture morphology of ultrasonic assisted Sn2.5Ag0.7Cu0.1RExNi/Cu solder joints under 500 thermal cycles. It can be seen from Fig. 14 that the shear fracture of solder joints was a ductile–brittle mixed fracture mode composed of parabola dimple and cleavages. With the increase of Ni content, the dimples in the fracture morphology become deeper, especially when the Ni content is 0.05wt%, the dimples in the solder joints are more and deeper. The above results showed that the addition of proper amount of Ni could inhibit the brittle transition of solder joints under thermal cycling. Figure 15 shows the XRD of the fracture morphology of ultrasonic-assisted Sn2.5Ag0.7Cu0.1RExNi/Cu solder joints under thermal cycling. With the increase in thermal cycles, the ratio of the (Cu,Ni)_6_Sn_5_ to β-Sn peak was higher. The results showed that the fracture path moved to the interfacial IMC side and it was no difference with the previous analysis. Figure 16 shows the schematics of the fracture model. For the samples (Fig. 6a) without thermal cycling, there were none microcracks and microvoids in the interfacial IMC. The fracture originated in interfacial transition zone and then propagated. However, the interfacial IMC layer of solder joints under thermal cycling was relatively thickness, and microcracks and microvoids were originated and grew in the interfacial IMC. This may lead to the stress concentration in the interfacial IMC due to the uncoordinated thermal expansion of various regions in the joints^24^. The crack initiated in the interfacial IMC and then propagated.

**Table 2:** EDS analysis results of “A”, “B” area in Fig. 13.

Area	Mole fraction/%			
	Sn	Cu	Ag	Ni
A	93.62	4.49	1.89	–
B	46.57	51.90	–	1.53

## Conclusions

In this study, the interfacial microstructure and shear properties of ultrasonic-assisted Sn2.5Ag0.7Cu0.1RExNi/Cu solder joints are investigated under thermal cycling (from – 40 to + 125 ℃) for up to 1000 cycles. Based on the resulted obtained, the following conclusions can be summarized:

1. Ultrasonic is an effective method to obtain high quality solder joints. When the ultrasonic vibration power is 88 W, the ultrasonic-assisted Sn2.5Ag0.7Cu0.1RE0.05Ni/Cu solder joints exhibits the optimized performance. (increases 29.1% comparing to the solder joints without ultrasonic).
2. The IMC layer of ultrasonic-assisted Sn2.5Ag0.7Cu0.1RExNi/Cu solder joints consisted of (Cu,Ni)_6_Sn_5_ and Cu_3_Sn layers. With the increase of thermal cycles, the IMC morphology of ultrasonic-assisted Sn2.5Ag0.7Cu0.1RExNi/Cu solder joints changed from "scallop" to "lamellar", the thickness and roughness of IMC increased, and the shear strength of solder joints decreased. Under the condition of 1000 cycles of thermal cycling, the thickness and roughness of IMC of solder joints with 0.05 wt% Ni was the smallest, and the shear strength of the solder joints was 19.8 MPa, which was 28.6% higher than that of the solder joints without Ni.
3. Ultrasonic can improve the thermal cycling reliability of solder joints. In the process of thermal cycling, the thickness of IMC of ultrasonic-assisted Sn2.5Ag0.7Cu0.1RExNi/Cu solder joints was linearly related to the square root of the equivalent time, which meant that the growth of IMC mainly occurred in the high temperature stage of thermal cycling. The growth of interfacial IMC of ultrasonic-assisted Sn2.5Ag0.7Cu0.1RExNi/Cu solder joints had an incubation period, and the growth of IMC was slow within 300 cycles. And after 300 cycles, the IMC grew rapidly, the granular IMC began to merge together, the thickness and roughness of IMC increased obviously, the defects such as microcracks and microvoids began to appear, and the shear strength of the solder joints decreased rapidly.
4. During the thermal cycling process, the shear strength of ultrasonic-assisted Sn2.5Ag0.7Cu0.1RExNi/Cu solder joints had a linear relationship with the thickness of IMC. With increasing the thermal cycling, the fracture mechanism of ultrasonic-assisted Sn2.5Ag0.7Cu0.1RExNi/Cu solder joints changed from ductile–brittle mixed fracture to brittle fracture. And the fracture pathway of ultrasonic-assisted Sn2.5Ag0.7Cu0.1RExNi/Cu solder joints changed from the transition zone of solder/IMC to the interfacial IMC.
